# Low-Cost, High-Efficiency Aluminum Zinc Oxide Synaptic Transistors: Blue LED Stimulation for Enhanced Neuromorphic Computing Applications

**DOI:** 10.3390/biomimetics9090547

**Published:** 2024-09-11

**Authors:** Namgyu Lee, Pavan Pujar, Seongin Hong

**Affiliations:** 1 Department of Physics, Gachon University, Seongnam 13120, Republic of Korea; kyle123456@gachon.ac.kr; 2 Department of Ceramic Engineering, Indian Institute of Technology (IIT-BHU), Varanasi 221005, Uttar Pradesh, India; 3 Department of Semiconductor Engineering, Gachon University, Seongnam 13120, Republic of Korea

**Keywords:** synaptic transistor, aluminum doped zinc oxide, solution process, large-scale production

## Abstract

Neuromorphic devices are electronic devices that mimic the information processing methods of neurons and synapses, enabling them to perform multiple tasks simultaneously with low power consumption and exhibit learning ability. However, their large-scale production and efficient operation remain a challenge. Herein, we fabricated an aluminum-doped zinc oxide (AZO) synaptic transistor via solution-based spin-coating. The transistor is characterized by low production costs and high performance. It demonstrates high responsiveness under UV laser illumination. In addition, it exhibits effective synaptic behaviors under blue LED illumination, indicating high-efficiency operation. The paired-pulse facilitation (PPF) index measured from optical stimulus modulation was 179.6%, indicating strong synaptic connectivity and effective neural communication and processing. Furthermore, by modulating the blue LED light pulse frequency, an excitatory postsynaptic current gain of 4.3 was achieved, demonstrating efficient neuromorphic functionality. This study shows that AZO synaptic transistors are promising candidates for artificial synaptic devices.

## 1. Introduction

Existing von Neumann computers face some bottlenecks, including poor parallel processing ability and high power consumption [1,2,3]. Attempts to overcome these challenges have led to the development of neuromorphic technologies, which mimic the efficient information processing of the human brain [4,5,6]. The human brain operates with a network of parallel-connected synapses, enabling learning, memorization, reasoning, and computation with a low power consumption of approximately 20 W [7,8,9]. Neuromorphic semiconductors function similarly as synapses, converting spike-shaped signals into electrical signals through synaptic devices [10]. Such devices have attracted attention in various fields, such as machine learning, artificial intelligence, and sensory information processing [11,12,13,14].

Current optoelectronic synaptic devices exist in various structures, such as two-terminal memristors and three-terminal transistors [3,15,16,17]. Compared with two-terminal memristors, three-terminal optoelectronic synaptic devices offer higher stability and the ability to apply various inputs, enabling self-learning [3,15]. Oxide semiconductors are transparent materials that are used in various devices and offer excellent versatility due to their capability for low-temperature processing [6]. Additionally, they exhibit low current leakage with high energy efficiency and can display various electrical properties such as those of semiconductors, insulators, and conductors, making them advantageous for designing a wide range of devices [5,14]. In addition, solution-based semiconductors are used in three-terminal optoelectronic synaptic devices, enabling large-area fabrication and excellent scalability [4,17]. In particular, solution-based oxide semiconductors exhibit high chemical stability and are inexpensive [17,18,19,20,21]. However, optoelectronic synaptic devices based on solution-based oxide semiconductors exhibit poor electrical and optoelectric properties because of defects in semiconductors [22]. Consequently, high-power optical stimuli or heterojunction semiconductor structures are used to enhance light absorption [14,23,24,25]. However, these methods are energy intensive, complicate the fabrication process, reduce stability, and introduce defects into the heterojunction interfaces, which hinder the implementation of synaptic plasticity [26].

In this study, we employed a solution-based aluminum-doped zinc oxide (AZO) as the semiconductor channel layer in the synaptic phototransistor. The presence of Al in ZnO enhances the charge concentration and may result in better electrical characteristics. The presence of Al^3+^ in the place of Zn^2+^ promotes the generation of additional electrons to maintain the electrical charge balance. The comparison of the electrical characteristics between the ZnO transistor and the AZO transistor is provided in the Supporting Information, Figure S1. The AZO channel layer can be fabricated on a large scale using a simple spin-coating method. A UV laser with a wavelength of 406 nm and blue LED light are absorbed by the AZO photoabsorption layer, and charge carriers are also transported through the AZO channel layer. The UV laser excites more electrons into the conduction band, thereby enhancing the EPSC response. In contrast, the blue LED operates with lower energy consumption, making it an efficient choice for driving synaptic devices. AZO exhibits synaptic responses under both UV laser and blue LED light, demonstrating versatility in adjusting the wavelength range to suit various applications. Doping with aluminum improves the conductivity of the AZO layer, and oxygen vacancies prevent electron recombination, resulting in excellent synaptic plasticity responses [27,28]. Therefore, the synaptic device fabricated herein effectively operates even under blue LED light and exhibits excellent synaptic behaviors, including short-term plasticity (STP), long-term plasticity (LTP), and excitatory postsynaptic current (EPSC). The solution-based AZO synaptic transistor is promising for cost-effective, efficient, and scalable neuromorphic devices. This technology can be applied in various fields that mimic synapses, providing a robust foundation for developing neuromorphic computing systems.

## 2. Materials and Methods

### 2.1. Solution Preparation

The AZO precursor solution was prepared by mixing aluminum nitrate nonahydrate (Al(NO_3_)_3_·9H_2_O) powder with zinc nitrate (Zn(NO_3_)_2_) powder at a concentration of 2 at.%. The mixture was dissolved in 0.2 M 2-methoxyethanol (≥99.9%, Sigma-Aldrich, St. Louis, MO, USA) and stirred at 400 rpm and 50 °C for 12 h, obtaining a transparent and clear AZO precursor solution without precipitates. Using zinc nitrate (Zn(NO_3_)_2_) powder and 2-methoxyethanol, a 0.2 M ZnO precursor was prepared, and the performance of devices with a ZnO channel was compared with that of the AZO channel devices.

### 2.2. Device Fabrication

A substrate with a 300 nm thick SiO_2_ on highly p^+^-doped Si was used. The SiO_2_ and Si layers act as the gate electrode and gate insulator, respectively. Before coating with the precursor solution, the substrate was cleaned with acetone, isopropyl alcohol, and deionized water using an ultrasonic cleaner. The cleaned substrate was then treated with O_2_ plasma for 1 min. Subsequently, the precursor solution was spin-coated onto the substrate at 3000 rpm for 20 s. To dry the solvent, the AZO-coated substrate was annealed at 300 °C for 15 min. Five AZO channel layers were produced by repeating the O_2_ plasma treatment, spin-coating, and annealing processes. Finally, the five-layer AZO-coated substrate was annealed at 300 °C for 2 h. Next, to deposit the electrodes, 60 nm of aluminum for ohmic contact and 40 nm of gold were deposited using a thermal evaporator. Finally, an AZO synaptic transistor was fabricated by patterning the electrodes through a lift-off process and the channels using a buffered oxide etchant.

### 2.3. Characterization

The surface and thickness of spin-coated AZO were investigated using an atomic force microscope (AFM) (AFM Workshop, TT-AFM) in the noncontact mode. The electrical and optoelectric properties of the synaptic transistor were measured in a dark box using a device parameter analyzer (Keysight, Santa Rosa, CA, USA, B1500A). Optical pulses were applied using a multichannel fiber-coupled laser source (Thorlabs, Newton, NJ, USA, MCLS1) and a digital storage oscilloscope (Fnirsi, Shenzhen, China, 1014D). Furthermore, the efficiency of the AZO synaptic transistor was measured using a blue LED and a microcontroller board (Uno, Gold Coast, QLD, Australia, R3). The appearance and configuration of the device were also measured using an optical microscope (Olympus, Münster, Germany, BX60). XPS (Thermo Fisher Scientific, Madison, WI, USA, NEXSA G2) and thin-film X-ray diffraction (XRD) (Bruker, Bremen, Germany, D8 DISCOVER) were employed to analyze the physical properties of AZO.

## 3. Results

### 3.1. AZO Synaptic Transistor Fabrication

**Figure 1:**
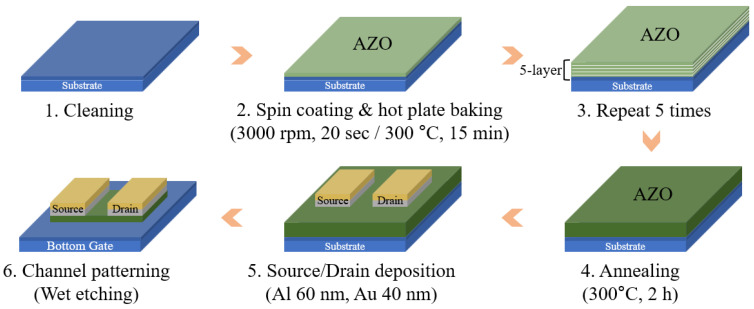
Schematic of the AZO synaptic transistor manufacturing method.

Figure 1 shows the manufacturing process of the AZO synaptic transistor. We adopted a back-gate, top-contact structure. The p^+^-doped Si and 300 nm thick SiO_2_ layer acted as the gate electrode and gate insulator, respectively. To eliminate residues, the substrate was subjected to ultrasonic cleaning with acetone, isopropyl alcohol, and deionized water. After cleaning, O_2_ plasma treatment was performed to enhance the coating properties of the AZO. Each layer was spin-coated and annealed at 300 °C for 15 min. The AZO channel layer was fabricated by repeating the spin-coating process five times, resulting in a sufficient channel thickness of 338.9 nm to ensure high conductivity [29,30]. To promote crystallization and improve the stability of the semiconductor channel layer, the substrate was annealed at 300 °C for 2 h using a hot plate. The annealing process is essential because it enhances charge carrier mobility [31,32,33]. Next, for ohmic contact, Al/Au electrodes (60/40 nm) were deposited using a thermal evaporator and patterned through a lift-off process. Finally, the AZO channel was etched using a buffered oxide etchant.

### 3.2. Effect of the Number of AZO Layers on the Surface Quality and Electrical Properties of the Transistor

**Figure 2:**
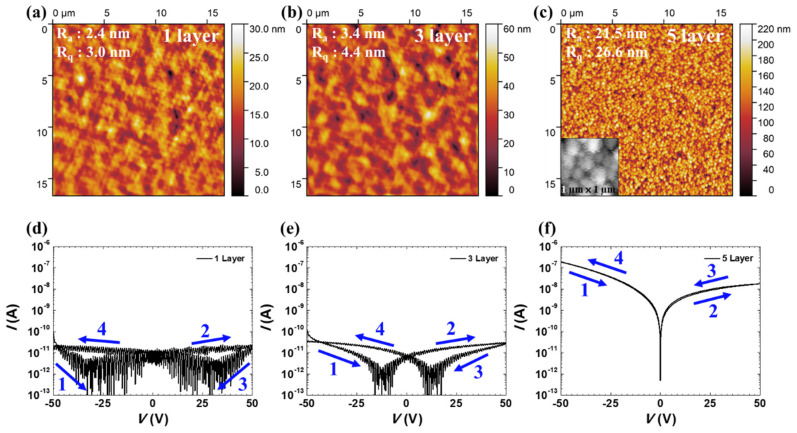
AFM images of the surfaces of (**a**) 1-, (**b**) 3-, and (**c**) 5-layer AZO. The inset in (**c**) shows an enlarged image (1 µm × 1 μm). The values in the images are the average and RMS roughness. *I*–*V* curves of (**d**) 1-, (**e**) 3-, and (**f**) 5-layer AZO devices.

Figure 2a–c shows AFM images of devices with different numbers of AZO layers, from which the surface density and roughness of AZO were evaluated. An area of 16.7 µm × 16.7 µm on the surfaces coated with different layers of AZO was scanned by the AFM. To evaluate the surface density of the layers, we measured the mean spacing of the profile irregularities (S_m_) to obtain the average distance between the surface irregularities. The S_m_ values were 978.5, 614.2, and 281.9 nm for the one-, three-, and five-layer AZO films, respectively. Furthermore, we evaluated the surface roughness of the films by measuring the roughness average (R_a_). The R_a_ values for the one-, three-, and five-layer AZO were 2.4 nm, 3.4 nm, and 21.5 nm, respectively. Additionally, film thicknesses of AZO are measured using the end-step method by AFM (Supporting Information, Figure S2). The thicknesses increased with the increase in the number of layers of coatings (from 1 layer to 5 layers). Table S1 shows the thickness values of the films. The thickness of the one-layer coating is 104.3 nm, the three-layer coating is 236.6 nm, and the five-layer coating is 338.9 nm. As the coating process progresses, it fills in fine surface defects, so the thickness does not increase linearly. The multi-layer coating was used to reduce pinhole formation and create a purer thin film. To further understand the surface roughness of the films, we measured their root mean square roughness (R_q_), which was 3.0 nm, 4.4 nm, and 26.6 nm for the one-, three-, and five-layer films, respectively. These results show that the surface density and roughness of the AZO thin films significantly increase with the number of coatings, indicating that crystallization is induced as the number of spin coatings increases. This is evident in the inset of Figure 2c, an enlarged image of the film (1 µm × 1 µm), which reveals that crystallization occurred in the five-layer film.

Figure 2d–f shows the effect of the AZO coating thickness on the electrical properties of the synaptic transistor. Additionally, the electrical characteristics of the sevenlayer AZO transistor are provided in the Supporting Information, Figure S3. If the AZO channel layer becomes too thick, it ceases to function as a semiconductor and instead exhibits conductive behavior. We measured the double-sweep *I*–*V* curves for different AZO layer thicknesses in the voltage range of −50 to 50 V. The blue arrow indicates the direction of the measured *I*–*V* curve. The increase in film volume may enhance the carrier concentration because of the high Al^3+^ concentration facilitated by oxygen vacancies [34]. Furthermore, as the thickness of the AZO layer increased, crystallization improved, resulting in a higher current level and stable operation due to the increase in the thickness of the semiconductor channel layer. These results indicate that the thickness of the AZO coating significantly affects the electrical properties of synaptic transistors.

### 3.3. Material Composition of the AZO Thin Films

**Figure 3:**
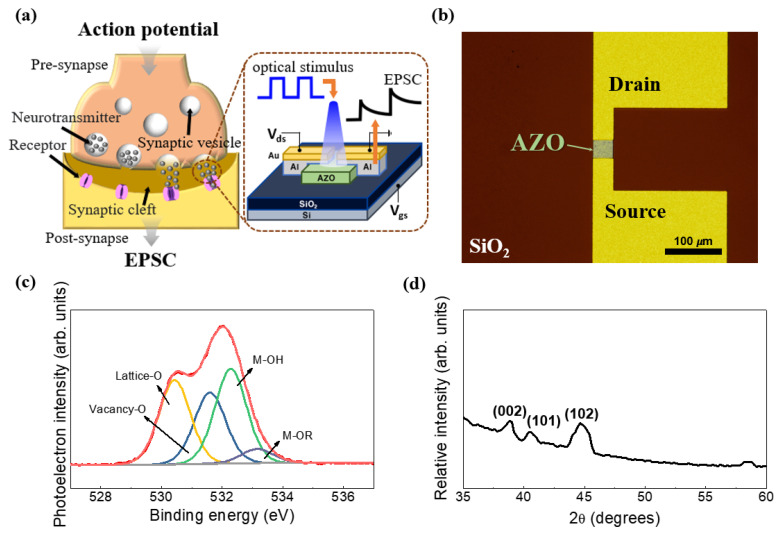
(**a**) Schematic of biological synapses and neural signal transmission in neurons and the AZO synaptic transistor, which mimics neural signal transmission in neurons. (**b**) Optical microscope image of the AZO synaptic transistor. (**c**) High-resolution O 1s spectrum of AZO, showing lattice oxygen, oxygen vacancies, and alkoxide impurities. (**d**) X-ray diffraction patterns of the thin films showing the amorphous nature of AZO.

Figure 3a shows a schematic of neural signal transmission in biological synapses and an AZO synaptic transistor that mimics this process. Synaptic plasticity involves an action potential reaching the nerve terminal, causing synaptic vesicles to fuse with the cell membrane [35,36]. This fusion releases neurotransmitters into the synaptic cleft, which stimulates postsynaptic neurons. Synaptic plasticity is the mechanism underlying various synaptic functions of the brain, such as learning and memorizing, and the strength of synaptic connections changes based on the frequency and pattern of activity in presynaptic and postsynaptic neurons [35,37,38,39]. In the developed AZO synaptic transistor, synaptic plasticity is induced by optical stimuli, resulting in EPSC generation. Figure 3b shows the optical microscope image of the AZO synaptic transistor. The channel width and length of the device were 37.9 and 33.2 µm, respectively. Furthermore, we performed an XPS analysis to evaluate the effect of the quality of the AZO film on its performance. The high-resolution O 1s signature peak was deconvoluted into various synthetic peaks, including lattice oxygen, hydroxide, alkoxide oxygen, and oxygen vacancy features. Significant amounts of lattice oxygen and vacancy oxygen were observed (Figure 3c), indicating that the film has free carriers, mainly electrons. Thus, the film is an n-type conducting oxide. Owing to its small thickness and doping concentration, it can be regarded as a semiconducting electronic-grade oxide [6,40,41]. The peak centered at low binding energy (~530.44 eV) in Figure 3c is attributed to lattice oxygen, and the peaks at higher binding energies of 531.6, 532.3, and 533.1 eV are attributed to oxygen vacancies, untransformed surface hydroxides (M–OH), and unreacted alkoxides (M–OR), respectively.

A previous study showed that amorphous oxide semiconductors exhibit better electrical properties than crystalline semiconductors [42]. Thus, we performed grazing incidence XRD analysis to determine the crystallinity of the AZO thin films. Figure 3d shows the XRD patterns of AZO coated on a Si/SiO_2_ substrate. Broad peaks are observed, indicating the initiation of crystallization, which is attributed to the annealing process. The formed crystallites were small; thus, the effective full-width half maxima were high. In addition, the patterns reveal a mixture of amorphous and crystalline phases.

### 3.4. Electrical and Optical Characteristics of the AZO Synaptic Transistor

**Figure 4:**
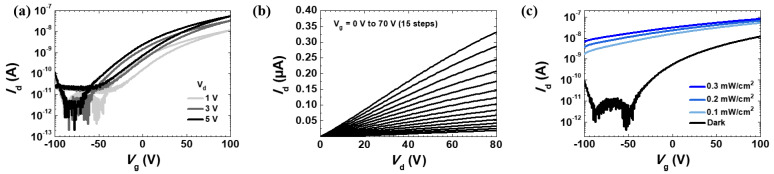
(**a**) Transfer curves of the 5-layer AZO synaptic transistor measured at drain voltages of 1, 3, and 5 V. (**b**) Output curves measured at gate voltages ranging from 0 to 70 V in 15 steps (5 V increments). (**c**) Forward-sweep transfer curves at a drain voltage of 1 V under dark and irradiation conditions. The incident power densities (*P*_inc_) of the UV light source were 0.1, 0.2, and 0.3 mW/cm^2^.

Figure 4a shows the transfer curves of the five-layer AZO synaptic transistor. The drain voltage (*V*_ds_) was set to 1, 3, and 5 V, and the gate voltage (*V*_gs_) varied from −100 to 100 V. The transfer curves show that the threshold voltage (*V*_th_) shifted toward negative bias, and the on current increased as the drain voltage increased. When the drain voltage is 1 V, the *V*_th_ is −47 V, the on/off ratio is 2.9 × 10^4^, and the subthreshold swing (SS) is 0.311 mV/dec. The reason for the threshold voltage having a large negative value is that the increased number of electrons from oxygen vacancies and aluminum doping causes electrons to accumulate more quickly in the channel. Figure 4b shows that the AZO synaptic transistor exhibits typical n-type characteristics. Figure 4c shows the transfer curves under UV light illumination (λ_ex_ = 406 nm) at *V*_ds_ = 1 V. UV light intensities of 0.1, 0.2, and 0.3 mW/cm^2^ were considered. As the UV light intensity increased, *V*_th_ decreased, and both the off and on currents increased significantly. This phenomenon occurs because UV light generates electron–hole pairs, which increase the charge density and conductivity in the channel. 

### 3.5. Synaptic Characteristics of the AZO Transistor

**Figure 5:**
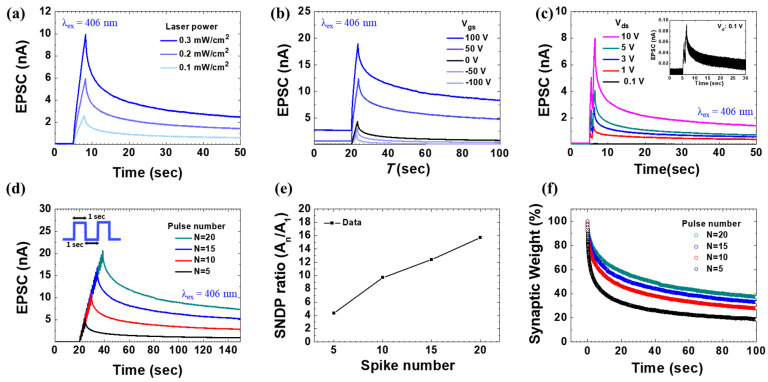
(**a**) Short-term potentiation (STP) to long-term potentiation (LTP) transition in the 5-layer AZO synaptic transistor with increasing *P*_inc_ (λ_ex_ = 406 nm, period (*T*) = 3 s, *V*_gs_ = 0 V, and *V*_ds_ = 10 V). (**b**) Variation in the excitatory postsynaptic current (EPSC) of the synaptic transistor with gate bias conditions (*T* = 5 s and *P*_inc_ = 0.3 mW/cm^2^). (**c**) EPSC modulation under different drain bias conditions at a pair of optical pulses (*T* = 1 s, *P*_inc_ = 0.1 mW/cm^2^ and *V*_gs_ = 0 V). The inset shows the modulation ar *V*_ds_ = 0.1 V. (**d**) EPSC modulation at different numbers of pulses (*T* = 2 s, *P*_inc_ = 0.2 mW/cm^2^, *V*_gs_ = 0 V, and *V*_ds_ = 10 V). (**e**) Variation in the SNDP ratio with the number of spikes. (**f**) Synaptic weight decay curves at different pulse numbers with normalized channel conductance at the final spike.

Short-term memory (STM) and long-term memory (LTM) in neuroscience are important concepts related to changes in the strength of connections between neurons through synaptic plasticity, specifically STP and LTP [43,44]. STP and LTP are distinguished by their postsynaptic responses, which vary with the degree of neurotransmitter release from the presynaptic neurons [45,46]. In an AZO synaptic transistor, neurotransmitter transmission is mimicked by optical stimuli, and a temporary increase in the postsynaptic response is represented by an increase in the postsynaptic current (PSC). STP, which plays a role in STM formation, involves a temporary increase in the postsynaptic neuron’s response due to an increased uptake of neurotransmitters, which rapidly returns to baseline once the stimulus ceases. Conversely, LTP, which plays a role in LTM formation, involves a long-lasting increase in synaptic strength due to the neuron’s enhanced ability to receive neurotransmitters. Figure 5a shows an increase in EPSC as the intensity of the optical pulses increased from 0.1 to 0.3 mW/cm^2^, indicating an STP–LTP transition. This indicates that as the intensity of the stimulus increases, the synaptic behavior changes from STP to LTP, mimicking the characteristics of biological synapses [47,48]. Because the fabricated synaptic device is a three-terminal device (i.e., transistor), the EPSC also changes with changes in *V*_gs_. Figure 5b shows the increase in EPSC as *V*_gs_ varied from −100 to 100 V. Compared with the case without gate bias, a negative gate bias suppresses EPSC, whereas a positive gate bias significantly enhances EPSC, demonstrating that both STP and LTP behaviors can be modulated by gate bias. Higher gate bias has more significant effects on potentiation. Negative *V*_gs_ causes electron accumulation at the surface of the AZO channel layer, preventing electron accumulation at the AZO/Al (semiconductor/metal) interface, thus suppressing EPSC. Conversely, positive *V*_gs_ promotes the accumulation of photogenerated electrons at the AZO/SiO_2_ (semiconductor/insulator) interface, significantly increasing EPSC [49,50]. Figure 5c shows the changes in EPSC as the drain voltage increases from 0.1 to 10 V under paired optical pulses at *T* = 1 s and *P*_inc_ = 0.1 mW/cm^2^. Higher drain bias resulted in higher spike amplitude and STP–LTP transition. The inset of Figure 5c shows increased synaptic plasticity even at a drain bias of 0.1 V. Thus, the STP and LTP behavior can be easily modulated by varying the operating voltage of the AZO synaptic transistor. Figure 5d shows the STP–LTP transition as the number of optical pulses increases from 5 to 20. Figure 5e shows the spike number dependent plasticity (SNDP) ratio, calculated as A_n_/A_1_, at varying numbers of spikes. The increased number of spikes mimics the increase in learning instances, which is reflected in the SNDP ratio. Figure 5f shows a decrease in synaptic weight with the increasing number of pulses. Synaptic weight is derived from normalized channel conductance, and more pulses result in a slower decrease in weight, indicating that increased learning instances reduce the rate of forgetting, similar to memory retention stages [51]. The high light intensity and short wavelength of the UV laser excite a large number of electrons in the AZO active layer, resulting in a strong photoresponse and demonstrating significant synaptic plasticity.

### 3.6. Synaptic Properties of Blue LED Light

**Figure 6:**
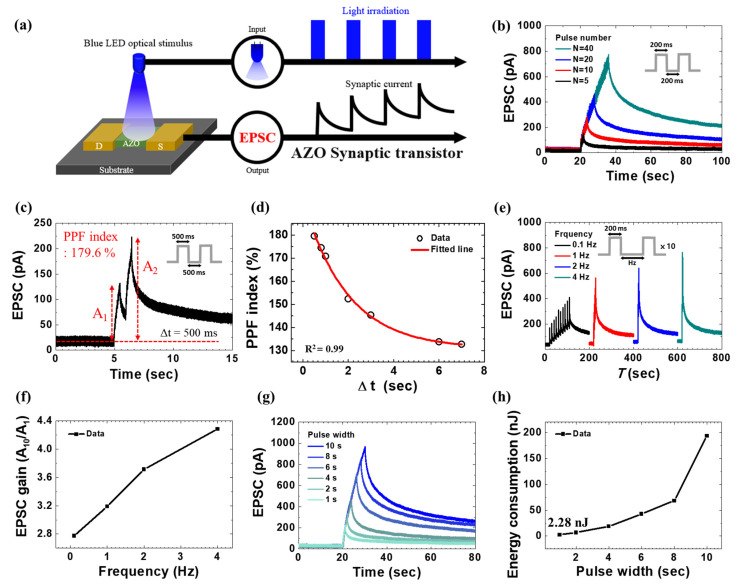
(**a**) Schematic of the AZO synaptic transistor operating with a blue LED. (**b**) EPSC (*V*_gs_ = 0 V, *V*_ds_ = 10 V, and *f* = 2.5 Hz) at different numbers of blue LED pulses, a presynaptic optical spike. (**c**) EPSC is induced by a pair of optical blue LED pulses at an interval time of 500 ms (*V*_gs_ = 0 V, *V*_ds_ = 10 V). (**d**) Paired pulse facilitation (PPF) index (*V*_gs_ = 0 V, *V*_ds_ = 10 V) as a function of optical pulse interval (Δt) with a pulse width of 500 ms. (**e**) EPSC (*V*_gs_ = 0 V, *V*_ds_ = 20 V) at a frequency of 0.1–4 Hz with a duration of 200 ms. (**f**) EPSC gain (A_10_/A_1_). (**g**) EPSC at different durations for a single pulse (*V*_gs_ = 0 V, *V*_ds_ = 10 V). (**h**) Energy consumption at different durations.

To efficiently operate synaptic transistors, it is essential to use light sources with suitable wavelengths, power efficiencies, and switching speeds [15]. To demonstrate the appropriate wavelength range, the results of EPSC measurements at various wavelengths (406 nm, 520 nm, 638 nm) are shown in Figure S4 (Supporting Information). If the wavelength exceeds the threshold, electrons in the valence band cannot be excited to the conduction band, preventing the device from functioning as an optoelectric synaptic device. Blue LEDs are suitable for illuminating synaptic transistors. LEDs have lower power consumption than lasers, which significantly reduces the power required to drive a synaptic transistor-based system. In addition, LEDs are highly scalable, making them adaptable to various systems. The fast switching time of LEDs allows for rapid light pulse modulation, thereby enabling efficient emulation of biological neurons. Moreover, LEDs are relatively inexpensive, reducing the development and production costs of AZO synaptic transistors and promoting cost-efficient neuromorphic computing. Figure 6a shows a schematic of an AZO synaptic transistor driven by a blue LED. The device operates by inducing synaptic currents (EPSC) when illuminated by blue LEDs. The EPSC increases with the number of blue LED pulses (Figure 6b), indicating that the AZO synaptic transistor mimics both STP and LTP under LED illumination. PPF is an indicator of synaptic connectivity strength. It is observed when the second stimulus elicits a greater postsynaptic response than the first. Figure 6c shows the EPSC of the fabricated transistor at *V*_gs_ = 0 V, *V*_ds_ = 10 V, and a pulse interval (Δt) of 500 ms. Table 1 is a performance comparison of oxide-based synaptic devices. Our research demonstrates that we have successfully created synaptic devices with superior performance compared to other studies, using a straightforward spin-coating method to fabricate the active layer, as evidenced by the PPF index. Further, the cycle-to-cycle and device-to-device reproducibility of the performance is described in Figure S5 (Supporting Information). The reproducibility and uniformity in the data show high thin film quality and robust AZO thin film. The PPF index is calculated as A_2_/A_1_ × 100, where A_1_ and A_2_ are the first and second amplitudes of EPSC, respectively [49,52]. The electron–hole pairs generated by the first pulse increase the PSC owing to the higher carrier density induced by the second pulse. Figure 6d shows the PPF index for Δt values ranging from 0.5 to 16 s. The PPF index decreased as Δt increased, and the fitted curve follows the function [53]:

$$PP\;Findex=A+C_{1}exp\left(\frac{-\Delta t}{\tau_{2}}\right)+C_{2}exp\left(\frac{-\Delta t}{\tau_{2}}\right),$$

where A is a constant, *C*_1_ and *C*_2_ are the initial magnitudes of the facilitation, representing rapid and slow phases, respectively, Δt is the interval between spikes, and *τ*_1_ and *τ*_2_ are the relaxation times for the rapid and slow phases, respectively. The fitted curve of the PPF index with adjusted R^2^ of more than 99%. Figure 6e shows the EPSC under blue LED illumination at frequencies *f* of 0.1, 1, 2, and 4 Hz. Each pulse sequence comprises 10 pulses with a width of 200 ms. As the frequency increased, EPSC induced by spikes also increased. Figure S6 (Supporting Information) shows the plot of EPSC vs. frequency depicting the consistency with minimum error. This indicates that synaptic transmission strength varies with frequency, mimicking the biological mechanism of adjusting neural connection strength for learning and information processing [54]. Figure 6f shows the ratio of the first spike (A_1_) to the tenth spike (A_10_). The EPSC gain increased significantly with frequency, indicating that higher frequencies promote synaptic enhancement, thereby improving signal transmission efficiency and emulating synaptic connectivity strengthening. Figure 6g shows the EPSC when the duration of a single pulse was increased from 1 s to 10 s at *V*_ds_ = 10 V. The magnitude of EPSC increased as the pulse duration increased, indicating an STP–LTP transition. This is because longer pulse durations generate more electron–hole pairs in the AZO channel, which accumulate in the channel. Figure 6h shows an increase in the power consumption of the AZO synaptic transistor with increasing pulse duration at a *V*_ds_ of 10 V, with a minimum power consumption of 2.28 nJ at 1 s. The AZO synaptic transistor shows a strong response under blue LED illumination, indicating efficient synaptic operation.

## 4. Conclusions

In this study, we fabricated synaptic transistors with a solution-based AZO channel layer that effectively mimics biological synapses. To efficiently mimic synaptic behavior, we used UV laser and blue LEDs as the light source, which effectively demonstrated synaptic plasticity. The results show that the UV laser demonstrates high responsiveness in inducing synaptic stimulation, while the blue LED can efficiently induce both STP and LTP with energy efficiency in AZO synaptic transistors. Thus, AZO synaptic transistors are suitable for high-efficiency and cost-effective neuromorphic computing devices, making them promising in various fields that require synapse-like functionality.

## Figures and Tables

**Table 1:** Performance comparison of oxide-based synaptic devices.

Active Layer	Active Layer Fabrication	PPF Index	Power Consumption	Reference
IGZO	Sputtering	~168% (Δt = 200 ms)	~1.1 pJ	[6]
IZO	Spin coating	~136% (Δt = 100 ms)	NA	[23]
IAZO	PLD	~155.9% (Δt = 200 ms)	~2.3 pJ	[28]
ZnO	Solution drop/RTA	~140% (NA)	~1 µJ	[55]
In_2_O_3_	Spin coating	~141% (Δt = 10 ms)	NA	[56]
AZO	Spin coating	~179.6% (Δt = 500 ms)	~2.28 nJ	This work

## Data Availability

The original contributions presented in this study are included in the article. Further inquiries can be directed to the corresponding authors.

## Supplementary Materials

The following supporting information can be downloaded at https://www.mdpi.com/article/10.3390/biomimetics9090547/s1, Figure S1: (a) Optical microscope image of the ZnO transistor. (b) Transfer curves of the 5-layer ZnO transistor measured at drain voltages of 1, 3, and 5 V. (c) Optical microscope image of the AZO transistor. (d) Transfer curves of the 5-layer AZO transistor measured at drain voltages of 1, 3, and 5 V; Figure S2: The AFM images show the step and corresponding thickness profile lines depicting the thickness of AZO films coated once, three times, and five times respectively; Table S1: The extracted thickness values of AZO thin films; Figure S3: (a) *I*–*V* curves of 7-layer AZO device. (b) Transfer curves of the 7-layer AZO synaptic transistor measured at drain voltages of 1, 3, and 5 V; Figure S4: (a) EPSC different wavelength conditions (UV = 406 nm, Green = 520 nm, Red = 638 nm) at a pair of optical pulses (*T* = 1 s, Pinc = 0.1 mW/cm^2^ and Vgs = 0 V). Inset image shows the EPSC when green light is applied. (b) Schematic diagram of electron excitation when green light is applied to AZO active layer. The reason for the response under green light will be explained in conjunction with Figure (b). The very faint EPSC response under green light is due to the excitation of a small number of electrons through subgaps caused by defects in the active layer, as shown in (1) of Figure (b). In most cases, the electrons excited into the subgap return to the valence band, as shown in (2); Figure S5: (a) EPSC modulation at UV pulses (N = 5, T = 2 s, Pinc = 0.2 mW/cm2, Vgs = 0 V, and Vds = 10 V). (b) EPSC modulation at UV pulses (N = 10, T = 2 s, Pinc = 0.2 mW/cm2, Vgs = 0 V, and Vds = 10 V). (c) EPSC induced 12 times by a pair of optical blue LED pulses at an interval time of 500 ms (Vgs = 0 V, Vds = 20 V); Figure S6: The variation of EPSC and energy consumption (in nJ) as a function of frequency and pulse width is depicted with minimum error.
