# Supplementary material for: Low-Cost, High-Efficiency Aluminum Zinc Oxide Synaptic Transistors: Blue LED Stimulation for Enhanced Neuromorphic Computing Applications
Source: Biomimetics (Basel). 2024 Sep 11;9(9):547. doi: 10.3390/biomimetics9090547 (PMC11430796; doi:10.3390/biomimetics9090547)
Supplement: Supplementary file 1 [file biomimetics-09-00547-s001.zip › biomimetics-3161027-supplementary.pdf]

Supporting Information

Low-Cost, High-Efficiency Aluminum Zinc Oxide Synaptic Transistors: Blue LED Stimulation for Enhanced Neuromorphic Computing Applications

Nam-Gyu Lee [1], Pavan Pujar [2,*], and Seongin Hong [1,*]

[1] Department of Physics, Gachon University, Seongnam 13120, Sujeong-gu, Republic of Korea; kyle123456@gachon.ac.kr

[2] Department of Ceramic Engineering, Indian Institute of Technology (IIT-BHU), Varanasi, Uttar Pradesh221005, India;

* Correspondence: pavan.cer@iitbhu.ac.in (P.P); seongin@gachon.ac.kr (S.H);

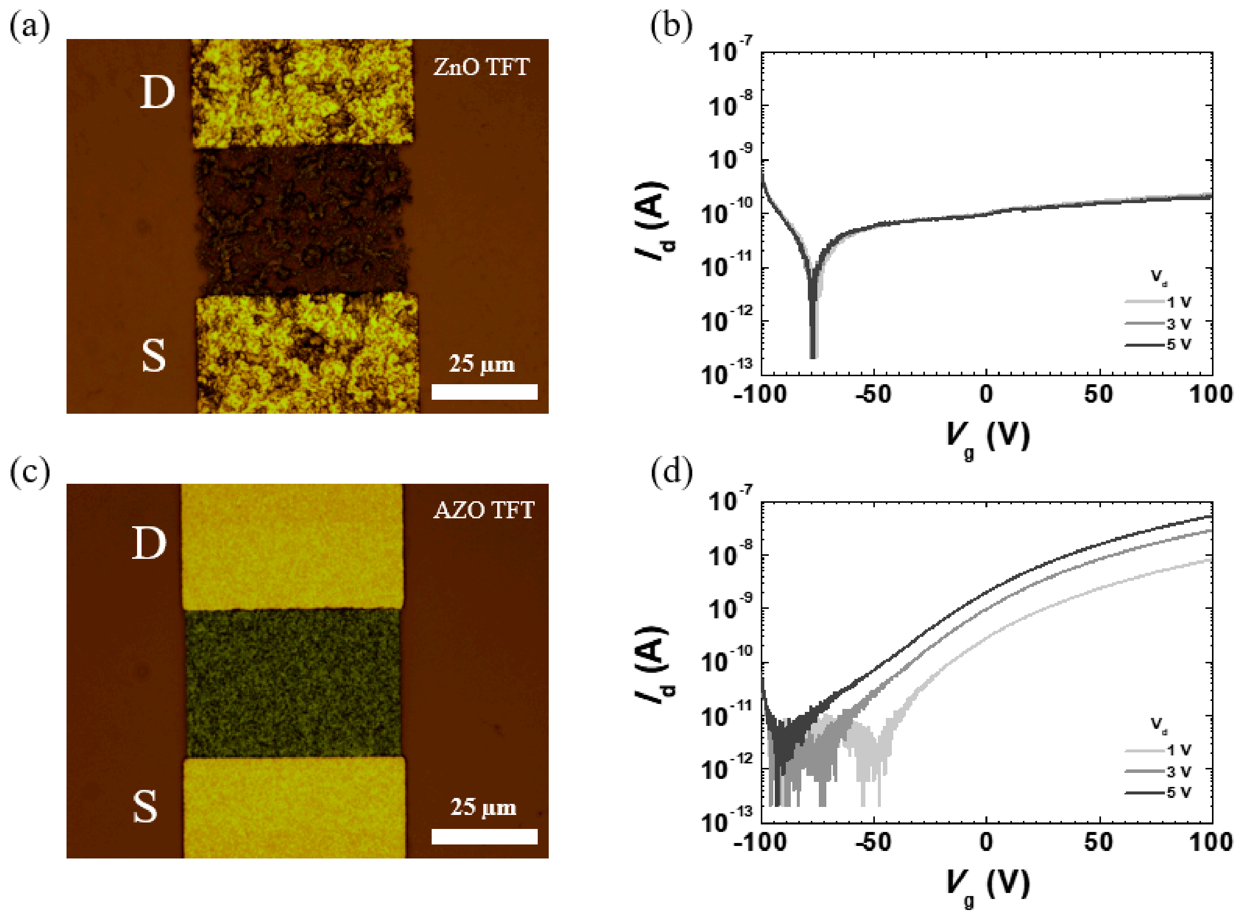

Figure S1. (a) Optical microscope image of the ZnO transistor. (b) Transfer curves of the 5-layer ZnO transistor measured at drain voltages of 1, 3, and 5 V. (c) Optical microscope image of the AZO transistor. (d) Transfer curves of the 5-layer AZO transistor measured at drain voltages of 1, 3, and 5 V.

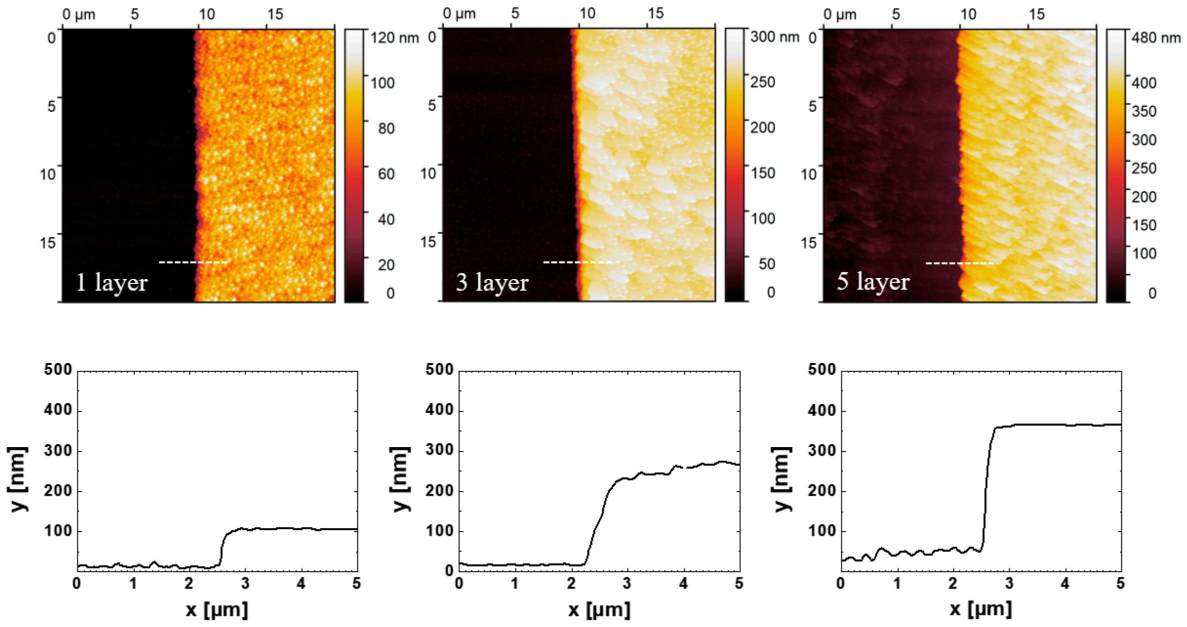

Figure S2. The AFM images shows the step and corresponding thickness profile lines depicting the thickness of AZO films coated once, three times and five times respectively.

Table S1: The extracted thickness values of AZO thin films.

Number of layers coated	Thicknesses in nm (measured using AFM)
1 layer	104.3 nm
3 layer	236.6 nm
5 layer	338.9 nm

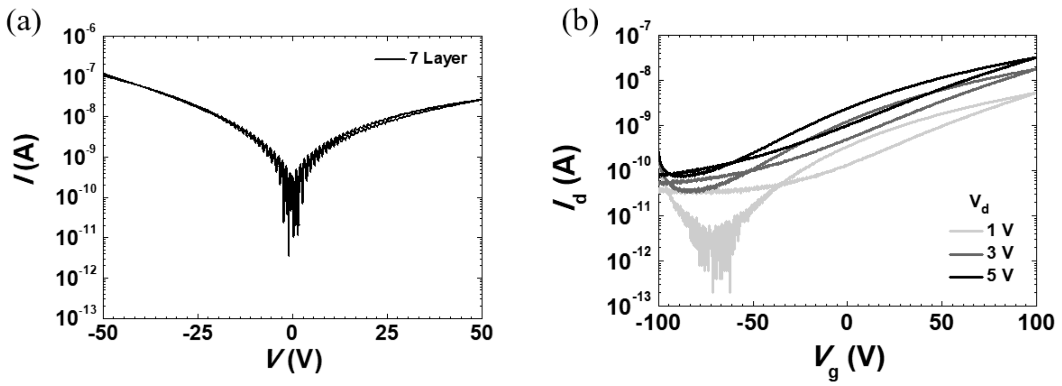

Figure S3. (a) I–V curves of 7 layer AZO device. (b) Transfer curves of the 7 layer AZO synaptic transistor measured at drain voltages of 1, 3, and 5 V.

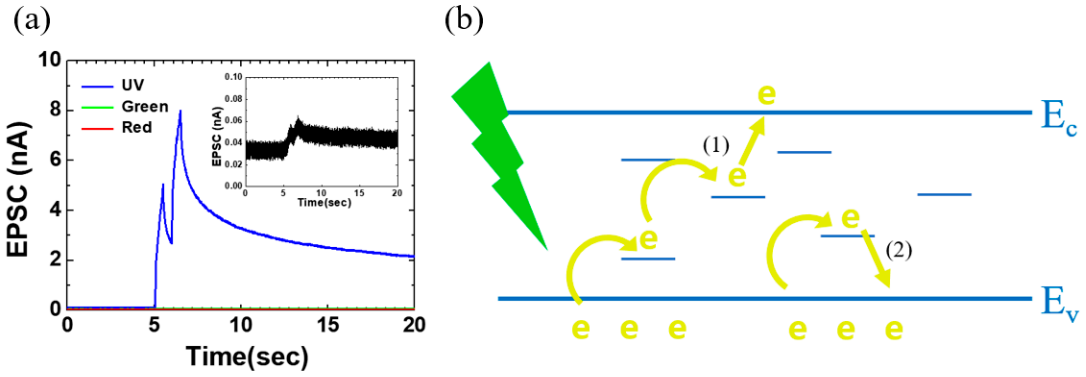

Figure S4. (a) EPSC different wavelength conditions(UV = 406 nm, Green = 520 nm, Red = 638 nm) at a pair of optical pulses (T = 1 s, P_{inc} = 0.1 mW/cm^2 and V_{gs} = 0 V). Inset image shows the EPSC when green light is applied. (b) Schematic diagram of electron excitation when green light is applied to AZO active layer. The reason for the response under green light will be explained in conjunction with Figure (b). The very faint EPSC response under green light is due to the excitation of a small number of electrons through subgaps caused by defects in the active layer, as shown in (1) of Figure (b). In most cases, the electrons excited into the subgap return to the valence band, as shown in (2).

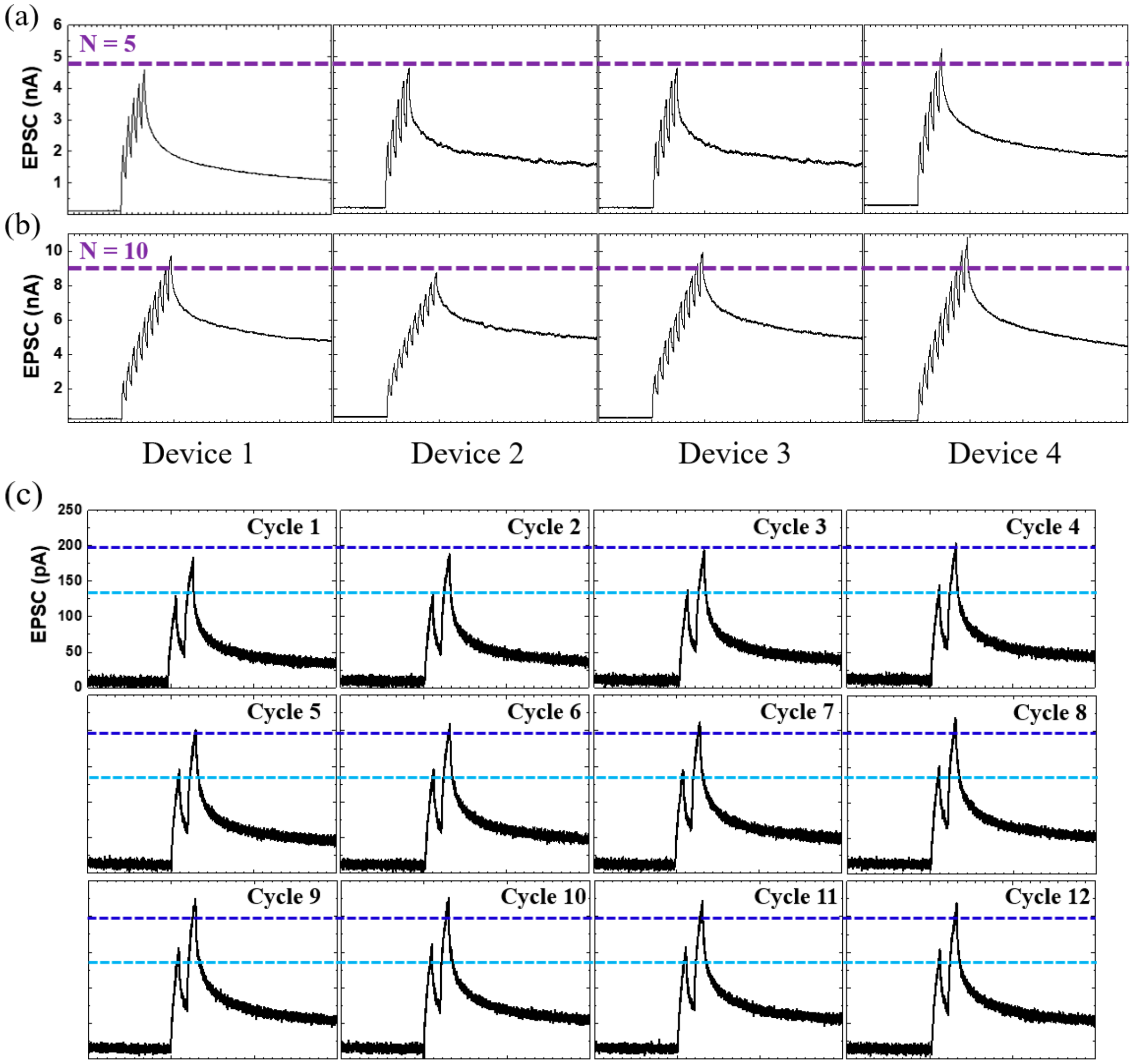

Figure S5. (a) EPSC modulation at UV pulses (N = 5, T = 2 s, P_{inc} = 0.2 mW/cm², V_{gs} = 0 V, and V_{ds} = 10 V). (b) EPSC modulation at UV pulses (N = 10, T = 2 s, P_{inc} = 0.2 mW/cm², V_{gs} = 0 V, and V_{ds} = 10 V). (c) EPSC induced 12 times by a pair of optical blue LED pulses at an interval time of 500 ms (V_{gs} = 0 V, V_{ds} = 20 V).

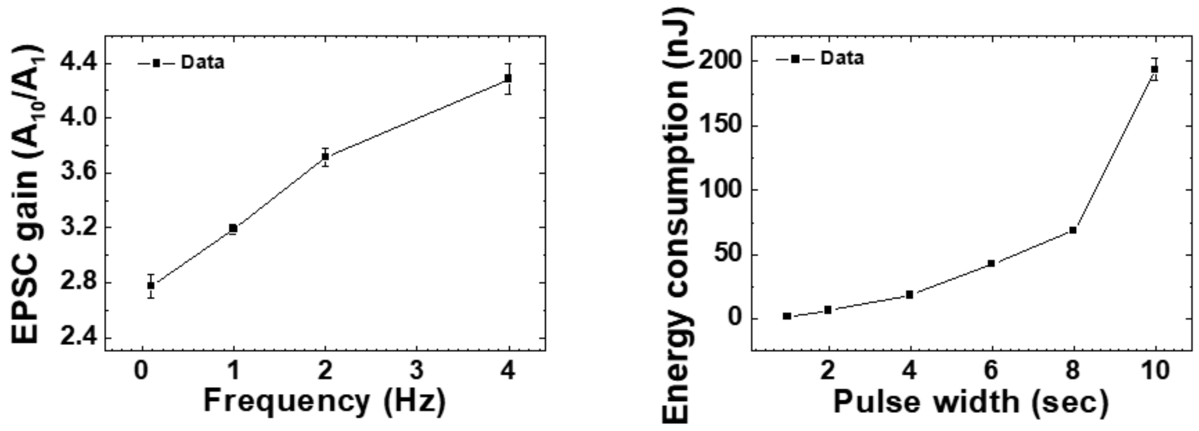

Figure S6. The variation of EPSC and energy consumption (in nJ) as a function of frequency and pulse width is depicted with minimum error.
